# The Role of the Emotional Sequence in the Communication of the Territorial Cheeses: A Neuromarketing Approach

**DOI:** 10.3390/foods11152349

**Published:** 2022-08-05

**Authors:** Vincenzo Russo, Marco Bilucaglia, Riccardo Circi, Mara Bellati, Riccardo Valesi, Rita Laureanti, Giuseppe Licitra, Margherita Zito

**Affiliations:** 1 Department of Business, Law, Economics and Consumer Behaviour “Carlo A. Ricciardi”, Università IULM, 20143 Milan, Italy; 2 Behavior and Brain Lab IULM—Neuromarketing Research Center, Università IULM, 20143 Milan, Italy; 3 Institute of Agricultural Biology and Biotechnology (IBBA), National Research Council of Italy (CNR), 20133 Milan, Italy; 4 Department of Management, Università degli Studi di Bergamo, 24129 Bergamo, Italy; 5 Departments of Electronics, Information and Bioengineering (DEIB), Politecnico di Milano, 20133 Milano, Italy; 6 Departmentf of Agricolture, Food and Enviroment (Di3A), Università di Catania, 95123 Catania, Italy

**Keywords:** emotions, neuromarketing, traditional cheese, territoriality, agri-food products

## Abstract

Over the past few years, many studies have shown how territoriality can be considered a driver for purchasing agri-food products. Products with certification of origin are perceived as more sustainable, safer and of better quality. At the same time, producers of traditional products often belong to small entities that struggle to compete with large multinational food corporations, having less budget to allocate to product promotion. In this study, we propose a neuromarketing approach, showing how the use of these techniques can help in choosing the most effective commercial in terms of likeability and ability to activate mnemonic processes. Two commercials were filmed for the purpose of this study. They differed from each other in terms of emotional sequence. The first aimed primarily at eliciting positive emotions derived from the product description. The second aimed to generate negative emotions during the early stages, highlighting the negative consequences of humans’ loss of contact with nature and tradition and then eliciting positive emotions by presenting cheese production using traditional techniques as a solution to the problem. Based on the literature on the emotional sequences in social advertising, we hypothesised that the second commercial would generate an overall better emotional reaction and activate mnemonic processes to a greater extent. Our results partially support the research hypotheses, providing useful insights both to marketers and for future research on the topic.

## 1. Introduction

Over the past few years, rootedness in the territory has become a very important motivational driver for the purchase of agri-food products [1,2,3,4]. In order to protect the rights of both consumers and producers, the European Union (EC Regulations 2081/92 and 2082/92) has identified three different designations that certify the products with a strong territorial identity: the Protected Designations of Origin, the Protected Geographical Indications, and the Traditional Specialities Guaranteed [5]. Those certified products are often associated with appealing concepts such as quality [6], tradition [7], sustainability [8], safety [2] and cultural identification [9]. Nevertheless, they struggle to compete with their industrial competitors in terms of budget allocation for communication campaigns since they are often produced by small and rural entities [10].

The communication campaigns make extensive use of emotions since they mediate and moderate consumer decision-making processes [11]. The effectiveness of commercials in generating emotions has been shown to be a good sales predictor [12]. In fact, emotions have a strong impact on message perception [13], increasing the likelihood that the advertised product or brand will attract attention and be remembered [14,15,16].

In recent years, much research has focused on how the sequence of opposite emotions (negative emotions followed by positive emotions or positive emotions followed by negative emotions) can be effective in persuading consumers [17]. Some evidence suggests that negative emotions followed by positive emotions are more effective because people perceive emotions based on an initial reference point [18,19]. For example, charity advertising most often tends to elicit negative emotions from the description of a problematic situation and then generate positive emotions from the description of the possibility of making actions to help people in need [20,21]. Although the issues and psychological mechanisms underlying the charity advertising are very different from those of agri-food products, similar concepts can be, in principle, applied to both. Just as the act of giving can help solve a problematic situation, the consumption of agri-food products rooted in the local area can be presented as a possible solution to problems such as pollution, communication asymmetry between consumer and producer, and the low perception of safety associated with industrial products [22,23,24,25].

In the past, the effectiveness of the communication campaigns in eliciting specific emotions had been studied mainly by assessing consumers’ conscious responses. However, the limitations of “classic” survey instruments used by marketers have been discussed in the literature for a long time now. Questionnaires [26,27], interviews [28,29] and focus groups [30] have been shown to be reliable only within certain limits, due to both the impossibility of obtaining detailed and/or truthful opinions from people [31] and the need to rely on subjective interpretations of interviewers that may not reflect the real internal dynamics of the consumer [30]. The lack of reliable methods for predicting consumer behaviour can have serious consequences: of the new products launched in the market, between 40% and 80% are doomed to fail, causing economic damage to companies quantifiable in the order of billions of dollars [32,33,34]. For these reasons, in recent years, there has been a growing interest in neuromarketing techniques [34,35].

We refer to neuromarketing as the use of neuroscience tools and insights to provide answers to challenges in business practices, especially in advertising and marketing research [36]. This discipline studies the latent mental processes underlying consumer behaviour [37,38]. The emergence of this strand of literature is due, on the one hand, to the need of marketers to identify methods that can better predict the success of marketing campaigns and, on the other hand, the need of neuroscientists to develop methods and techniques that can increase our knowledge of the brain [34,39,40,41]. Neuromarketing aims to overcome the limitations of traditional marketing methodologies by directly investigating emotional reactions using tools capable of detecting electrophysiological variables [40,41,42,43].

Neuromarketing applications are widespread. They include the evaluation of static advertising in both digital and printed format [44], radio and video commercials [45], as well as product packaging [46]. In addition to the profit-making sector, no-profit organisations operating in the charity [47] and social utility [48] have taken advantage of neuromarketing. Within the food and beverage sector, neuromarketing investigated the effectiveness of the food packages in communicating key factors such as the health content of the labels and the presence of additives [49], as well as the consumption sustainability [50] and the territoriality [1].

To the best of our knowledge, no study has ever assessed the effectiveness of certified agri-food product communication using neuromarketing techniques. In addition, no study has ever investigated the role of the emotional sequence in the communication of certified agri-food products.

This study fills these gaps in the literature, evaluating the emotional impact of two different video commercials created to promote certified cheeses from Southern Italy. Both the commercials focused on themes such as references to territory, production techniques and natural landscapes as key communication drivers. References to territoriality are often used because the specificity of the area of origin and the limited production area help endow the product with special characteristics in the eyes of the consumer [51]. Emphasis on production techniques was placed to emphasise sustainability in terms of respect for the environment and support for local people [22,23]. Finally, since certified products are also characterised in terms of eco-sustainability and environment preservation, references to nature are often present in their promotion [52].

Although the two videos focused on the same themes, they differed in terms of emotional sequence. The first, named “Rewind”, was designed to elicit mostly positive emotions, focusing mainly on aspects related to the goodness of the product and production techniques. The second, named “The Myth”, was designed to elicit an emotional sequence from initial negativity to positivity in the end: the theme of the territoriality is proposed as a solution to the problems represented by the loss of contact of humans with territories and traditions. A detailed description of the videos can be found in Appendix A.

As common practice in neuromarketing studies [53], we collected three electrophysiological signals: the electroencephalogram (EEG), the skin conductance (SC) and the photoplethysmogram (PPG). Four different indices were calculated from the above-mentioned signals:

- Approach-Withdrawal Index (AWI): an EEG-based index associated with the instinctive reaction of approaching towards or moving away from a stimulus [54,55];
- Memorisation Index (MI): an EEG-based index associated with successful memory encoding [56,57];
- Hearthrate (HR): a PPG-based index associated with the emotional valence [58]
- Emotional Index (EI): a compound SC- and PPG-based index that summarises the emotional degree (either positive or negative) [59].

We compared both the overall videos and the sequences corresponding to the four narrative themes (i.e., territory, product, production techniques, natural landscapes) to explore the impact of the emotional sequence on the affect (AWI, HR and EI indices) and the memorisation (MI index).

This study was intended to help producers identify the most effective communication strategy for territorial agri-food products. We believe it represents an added value, especially for small local realities, helping them to optimise investments and reduce the gap with the big food corporations.

## 2. Materials and Methods

### 2.1. Research Hypotheses

In the text below, we referred to the videos “Rewind” and “The Myth”, as, respectively, “R” and “M”. The themes of “territory”, “product”, “production techniques” and “natural landscapes” were shortened to, respectively, “Tr”, “Pr” “Prd” and “Nt”.

The general aim of the study was detailed in terms of the following six research Hypotheses (H1)–(H6):

**Hypothesis** **1 (H1).** *The video characterised by a negative–positive emotional sequence (M) generates, overall, a more positive emotional reaction than the commercial predominantly focused on positive emotions (R). We expect greater AWI, EI and HR values in M than in R;*

**Hypothesis** **2 (H2).** *The themes Tr, Prd, and Nt will generate a more positive reaction in video M than R. For each theme, we expect greater AWI, EI and HR values in M than R;*

**Hypothesis** **3 (H3).** *Pr sequences generate a more positive emotional reaction in M than in R. We expect greater AWI EI and HR values in M than in R;*

We also expect an impact on the salience, and thus on their memorisation, of the elements of the emotional sequence. Therefore, we hypothesised that:

**Hypothesis** **4 (H4).** *The video M activates greater memorisation processes than R. We expect greater MI values in M than R.*

**Hypothesis** **5 (H5).** *The themes Tr, Prd, and Nt will activate greater memorisation processes in M than R. For each theme, we expect greater MI values in M than R;*

**Hypothesis** **6 (H6).** *Pr activates greater memorisation processes in M than R. We expect greater MI values in M than in R.*

### 2.2. Instrumentation

We recorded the EEG using an NVX-52 device (Medical Computer Systems, Ltd., Moscow, Russia) at a sample frequency of 2 kHz and a resolution of 24 bits. We placed 38 active Ag/AgCl electrodes on the scalp according to the 10–20 system [60] by means of an elastic cap, in addition to two Ag/AgCl earlobes electrodes and one Ag/AgCl adhesive patch that served, respectively, as reference and ground.

We recorded the SC and the PPG signals using, respectively, the GSRSens (Medical Computer Systems, Ltd.) and FpSens (Medical Computer Systems, Ltd.) sensors, both connected to the auxiliary inputs of the NVX-52. We placed the two Ag/AgCl electrodes of the GSRSens on the index and ring finger from the non-dominant hand and the FpSens on the middle finger from the same hand. Both the GSR and PPG signals were acquired synchronously to the EEG at the same sample frequency and resolution. The recordings were controlled by the NeoRec software (Medical Computer Systems, Ltd.).

We used the iMotions software (iMotions, A/V) to deliver the stimuli. At the beginning of the experiment, iMotions generated a TTL pulse that was fed into the digital inputs of the NVX-52 using the ESB (EEG Synchronisation Box) [61]. This served to perform an off-line synchronisation between the recorded data and the stimuli timestamps.

### 2.3. Study Population and Experimental Protocol

Forty healthy Italian subjects (20 males) with ages ranging from 33 to 56 years (M = 45.67, SD = 7.36) were enrolled in the experiment. The subjects were randomly divided into two sub-groups of 20 subjects each. The groups did not differ in terms of mean age and gender proportions, as verified by the Mann–Whitney (W = 200.500, *p* = 1.000) and chi-squared ($\chi^{2}\left(1\right)=0.000,p=1.000$) tests, respectively.

The sample size was selected after a sensitivity analysis that was performed using the G*Power software [62] with the following input parameters:

- α=0.05;
- (1−β)=0.95;
- Total sample size = 40;
- Number of groups = 2;
- Correlation among the repeated measures = 0.5;
- Nonsphericity correction = 1.

The computed effect size was f=0.235, corresponding to a medium value [63].

The study protocol followed the Helsinki declaration and informed written consent was obtained from each participant.

Each subject sat on a chair placed in front of a 23.8-inch monitor (FlexScan EV2451, Eizo KK) located in a 7 × 3 m experimental room, artificially lit by florescence lights and in the absence of any natural light. Two experimenters positioned the SC, PPG and EEG sensors and checked the quality of the signals before starting the recording. The contact impedance of the EEG electrodes was measured and ensured to be less than 10 kΩ [64].

At the beginning of experiment, the subject performed a 60-s-long eye-closed baseline (EYC), followed by a 2-min-long neutral baseline (BSL). Then, according to the group splitting, either the M or R video was proposed.

### 2.4. Video Segmentation

For each video, the sequences corresponding to the 4 narrative themes (Nt, Tr, Pr, Prd) were identified and manually marked by 2 independent judges using the Boris software [65]. In order to compute the inter-rater reliability, the Cohen’s κ was evaluated within a 2s-long sliding window. We obtained values of κ=0.83, and κ=0.86 for video M and R, respectively, corresponding to a strong agreement [66]. Onsets and durations of the themes were built as the intersection between the chunks identified by the two raters. Finally, the onsets and the durations of both the EYC and BSL epochs, as well as the 4 themes, were exported for the subsequent analyses.

### 2.5. EEG Processing

The EEG was processed using Matlab (The Matwhorks, Inc., Natick, MA, USA) and the EEGLab toolbox [67], following a previously proposed standard pipeline [54,68].

First, the data were re-referenced to the linked earlobes and down-sampled to 512 Hz. Then, a band-pass filter (0.1–30 Hz) and a notch filter (50 and 100 Hz) were applied in order to remove the physiological and external noise. The Artefact Subspace Reconstruction (ASR) with a default cut-off parameter (k = 20) was applied in order to remove non-stationary artefacts [69]. The data were then decomposed into Independent Components (ICs) using the SOBI algorithm [70]. By using the neural-net based classifier ICLabel [71], artefactual ICs were identified as those with brain probability Pr{brain} ≤0.7 and set to zero, while non-artefactual ICs were back-projected to the original sensor space. A Current Source Density (CSD) reference was then applied in order to increase the spatial resolution of the EEG at the sensor level [72].

Finally, the cleaned EEG was epoched according to the onset and the duration of the EYC and BSL stimuli, as well as the narrative sequences. For each subject, we computed the Individual Alpha Frequency (IAF), which is defined as the centre of gravity of the PSD within the extended alpha range (7.5–12.5 Hz) [73]. In the IAF calculation, we considered, as PSD, the mean PSD aver-aged across all the occipital channels. The occipital PSDs were computed using the EYC data. Finally, we computed 2 canonical EEG bands as: δ=[0;IAF−6] Hz and α=[IAF−2;IAF+2] Hz [74].

In order to have the highest temporal resolution, all indices were computed following the filtering approach, which is based on filtering and averaging an appropriate set of EEG channels to produce a cluster [54]. The Hilbert Transform was applied to the filtered channels before the averaging to compute the smoothed instant power [75]. The AWI was obtained by subtracting the α-filtered right-frontal (FP2, F4, F8, FT8, FC4) and left-frontal (FP1, F3, F7, FT7, FC3) clusters [54], while MI was obtained as the θ-filtered left-frontal (FP1, F3, F7, FT7, FC3) cluster [57].

### 2.6. SC and PPG Processing

The SC and PPG signals were processed using Matlab (Mathworks, Inc.), following a previously proposed standard pipeline [68,76].

The SC signal was first band-pass filtered (0.001–0.35 Hz); then, a threshold for SC extreme values (0.05–60 μS) and extreme rate of changes (±8 μS/s) was used in order to detect artefacts [77]. The artefactual points were replaced by a linear interpolation using adjacent points. From artefact-corrected SC, the tonic Skin-Conductance Level (SCL) was extracted by means of the cvxEDA algorithm [78].

The BVP signal was first low pass filtered (5 Hz); then, all peaks were identified using the Pan–Tompkins algorithm [79], and the instant HR was computed from the inverse of the peak-to-peak distance. Finally, the HR signal was linearly interpolated and filtered with a 2s-long moving average filter in order to obtain a smoother signal.

By means of a trigonometric transformation, SCL and HR were converted into the uni-dimensional EI [57].

### 2.7. Baseline Normalisation

AWI, MI, HR and EI signals were epoched according to the narrative sequences and z-score transformed with respect to the BSL as [76]:

(1) $$x^{\prime }\left(t\right)=\left[x\left(t\right)-m_{BSL}\right]/s_{BSL}$$

where $x^{\prime }\left(t\right)$ is the z-score transformed signal, x(t) is the original signal, $m_{BSL}$ is the temporal mean of x(t) in the BSL epoch and $s_{BSL}$ is the temporal standard deviation of x(t) in the BSL epoch.

Then, the signals were temporally averaged across each narrative sequence in order to obtain a condensed stimulus-related index [80]. For each Video × Theme combination, outliers were identified by means of the inter-quantile range (IQR) criterion as points outside the interval $\left[Q1-1.5\times \mathrm{IQR};\;Q3+1.5\times \mathrm{IQR}\right]$, where Q1 is the first quartile, Q3 is the third quartile and IQR=Q3−Q1 [81].

### 2.8. Statistical Analyses

The statistical analyses were performed using JASP v.0.14 [82]. Each index was analysed by a two-way mixed ANOVA, considering the Video as a between-subject factor (two levels: M, R) and the narrative theme (hereinafter, Theme) as the within factor (four levels: Nature, Territory, Product, and Production). Prior to the analyses, the sphericity of the Theme and the equality of variances of the Video were assessed by the Levene’s and Mauchly’s tests, respectively. In the case of sphericity violations, the Greenhouse-Geisser correction based on the sphericity estimator ω was applied [83]. All the post-hoc comparisons were Holm-corrected. In the following section, all the significant differences were provided either as mean (M) and standard deviation (SD) or marginal mean (MM) and standard error (SE).

After the processing phase, 3 subjects were excluded from further analysis due to the excessive noise in their physiological signals. The final sample consisted of 37 subjects (19 males) with ages ranging from 33 to 56 years (M = 45.24, SD = 7.48). The M and R subgroups groups still did not differ in terms of mean age and gender proportions, as verified by means of the Mann–Whithney (W = 198.500, *p* = 0.411) and chi-squared $(\chi^{2}\left(1\right)=0.026$, *p* = 0.873) tests, respectively.

## 3. Results

### 3.1. EEG-Related Indices

**Figure 1:**
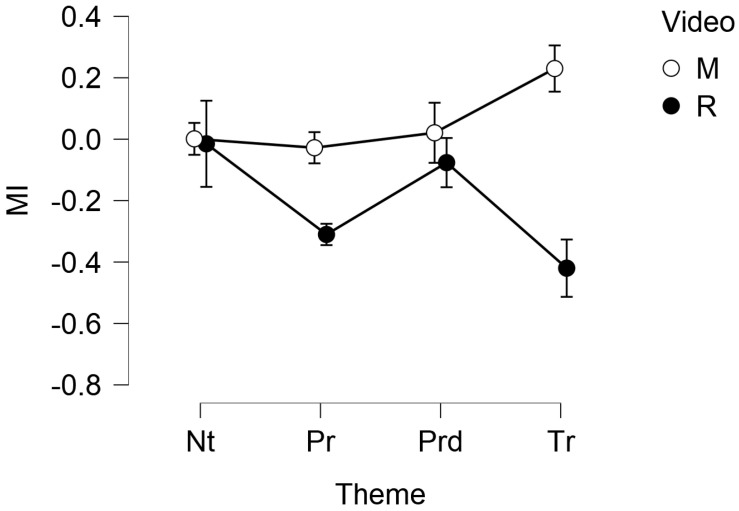
Descriptive plot with error bars of the MI, split for the video (M = The Myth, R = Rewind) and the theme (Nt = Nature, Pr = Product, Prd = Production, Tr = Territoriality). The vertical axis is expressed as unit-less z-scores.

The AWI did not show any significant main effect or interactions. The MI showed a significant main effect for the video (F(33,1)=5.493,p=0.025) and a significant interaction of the theme × video (F(2.250,74.246)=5.711,p=0.004,ω=0.750). Post-hoc comparisons showed a significant (p=0.025) difference between M (MM=0.060,SE=0.079) and R (MM=−0.202,SE=0.079). Nature × R (M=0.001,SD=0.251) and Territory × R (M=−0.420,SD=0.480) showed a significant (p=0.027) difference, similarly to Product × R (M = −0.310, SD = 0.365, n = 17) and Territory × M (M = 0.230, SD = 0.481) - *p* = 0.015, as well as Territory × M (M = 0.230, SD = 0.481) and Territory × R (M = −0.420, SD = 0.480) - *p* = 0.001. The following Figure 1 and Table 1 show, respectively, the descriptive plot with standard error bars and the descriptive statistics of the MI, split for video and theme.

### 3.2. SC- and BVP-Related Indices

**Figure 2:**
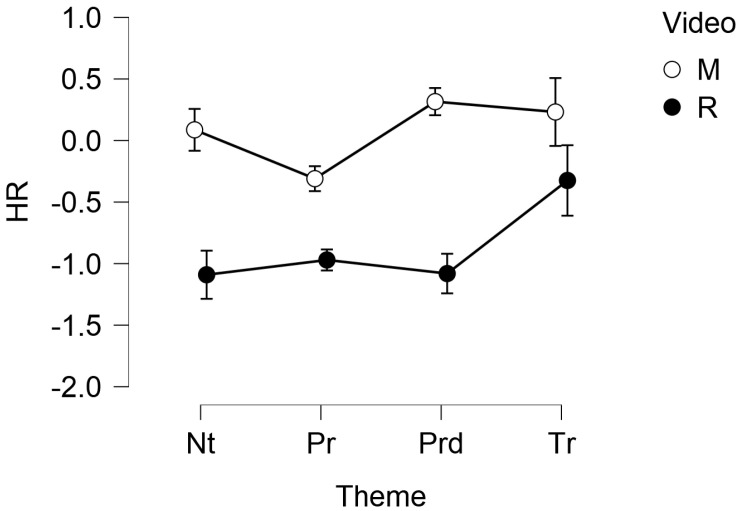
Descriptive plot with error bars of the HR, split for the video (M = The Myth, R = Rewind) and the theme (Nt = Nature, Pr = Product, Prd = Production, Tr = Territoriality). The vertical axis is expressed as unit-less z-scores.

HR showed a significant main effect of the theme (F(1.504, 45.135) = 3.669, *p* = 0.045, ω=0.501) and the video (F(1, 30) = 15.263, *p* < 0.001). Post hoc comparisons found a significant (*p* = 0.013) difference between Product (MM = −0.640, SE = 0.167) and Territory (MM = −0.046, SE = 0.167), as well as between M (MM = 0.081, SE = 0.172) and R (MM = −0.867, SE = 0.172), *p* < 0.001. The following Figure 2 and Table 2 show, respectively, the descriptive plot with standard error bars and the descriptive statistics of the HR, split for video and theme.

**Figure 3:**
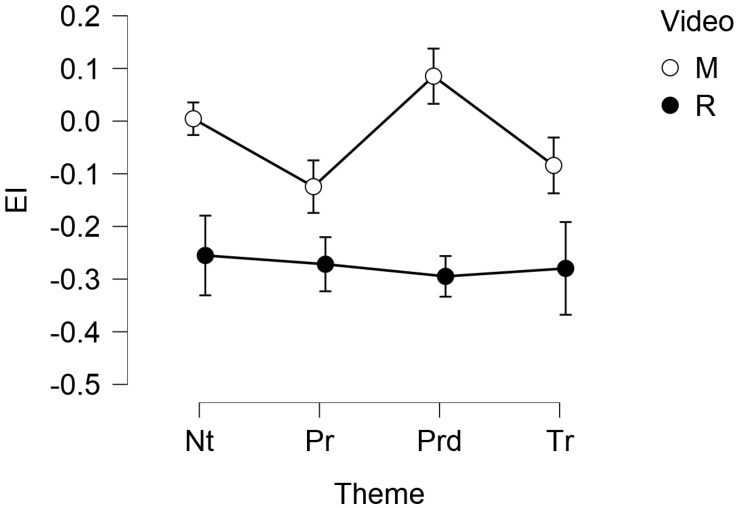
Descriptive plot with error bars of the EI, split for the video (M = The Myth, R = Rewind) and the theme (Nt = Nature, Pr = Product, Prd = Production, Tr = Territoriality). The vertical axis is expressed as unit-less z-scores.

EI showed a significant main effect of the video (F(1, 31) = 7.728, *p* = 0.009). Post hoc comparisons showed a significant difference between M (MM = −0.033, SE = 0.062) and R (MM = −0.279, SE = 0.062), *p* = 0.009. The following Figure 3 and Table 3 show, respectively, the descriptive plot with standard error bars and the descriptive statistics of the EI, split for video and theme.

## 4. Discussion

In this study, we investigated the role of emotional sequence in the communication of traditional cheeses from Southern Italy. For this purpose, we compared several physiological indices (AWI, MI, HR and EI) of two groups of participants during the vision of two video commercials. The first group watched a video (R) mainly characterised by a positive emotional tone, with sequences focused on the product quality and the traditionality of production processes. The second group watched a video (M) characterised by initial negative emotions, elicited by sequences showing the consequences of losing contact with the territory and traditions, followed by positive emotions, obtained by showing the positive consequences of regaining contact with the traditions and the territory. The videos were segmented by two individual raters into four narrative themes (Nt, Pr, Prd and Tr), and the physiological indices were averaged across the duration of each theme. We advanced six research hypotheses (H1–H6) that compared several metrics (AWI, MI, HR and EI) across both the Video and the Video × Theme dimensions, as summarised in the following Table 4.

The Video M showed, overall, greater EI and HR than R, while AWI did not show any significant difference. This partially supports the research hypothesis H1, which assumed a greater emotional reaction in the emotional sequence. The AWI results must not be read as a contradiction to those of EI and HR for at least two reasons. First, despite the fact that EI, HR and AWI can be associated with the same psychological construct of the emotional valence, they belong to different divisions of the nervous system: the autonomous nervous system (ANS) for EI and HR, and the central nervous system (CNS) for the AWI [53]. It was shown that these sub-systems are non-linearly related, and the degree of their coupling linearly depends on other factors, such as the levels of arousal of the emotionally-relevant stimuli [84]. Since the storytelling and the framing of the videos were not designed to elicit high levels of arousal, a low coupling between the ANS and CNS measures is expected. Second, some studies have questioned the appropriateness of the AWI as a measure of emotional valence [85]. Despite the fact that people are generally attracted to what elicits positive emotions and tend to turn away from what elicits negative emotions, it is also true that not all negative emotions cause a turning-away reaction. Anger, for example, despite being a negative emotion, generates an instinctive approach response [86]. Within the negative emotions of Video M, it is likely to expect the presence of anger, especially in the sequences related to the men’s loss of contact with territories and traditions, as well as to the Godhead’s punishment. The insignificant main effect of the Video could be, thus, due to the comparison of two positive AWI values, one associated with positive emotions and the other with anger.

The differences between M and R on EI, HR and AWI values related to Tr, Prd, Pr and Nt themes did not reach statistical significance, not supporting H2 or H3. A possible explanation could be related to the difference in the storytelling between the two videos that, according to past studies [87,88], has a strong role in mediating and/or moderating the emotional content of the video commercials. In statistical terms, the storytelling may have played the role of a confounding factor in decreasing the effect size associated with the interactions, leading to non-significant differences across the themes. This should be verified with a future confirmatory study based on stimuli with fixed storytelling but variable emotional sequence.

Compared to R, M showed an overall significantly greater MI, fully supporting H4, which assumed a different impact of the videos on the salience and, thus, the memory encoding. This is in line with previous researches on charity advertising that underlined the role of the emotion sequences in enhancing the overall salience [20,21]. Salience, in turn, plays a key role in the memorisation process: it was shown that maximal-saliency stimuli are associated with a greater recollection probability, and they facilitate access to memory representation at retrieval [89].

The M video showed a significantly greater MI than R only for the Tr theme, only partially supporting H5, which assumed greater memorisation of Nt, Prd and Tr themes in M. For the Pr theme, MI did not show a significant difference between M and R: this did not support H6, which assumed a greater memorisation in Video M. Similarly to what was discussed with H2 and H3, the different storytelling could have played a confounding role since, according to past studies [87,90], it also has a strong impact on the memorisation processes.

There is a chance that some research hypotheses have been rejected due to the characteristics of the sample, rather than the feature of the videos. In fact, it has been shown that gender and age play a significant role in emotional evaluation [91] and episodic memory recall [92]. A confirmatory study based on a four-way mixed ANOVA design with gender and age as additional between-subject factors is suggested to verify this supposition.

It is worth mentioning the limitations of the present study. We evaluated two videos that had never previously aired since they were shot specifically for this study. Additionally, the two creative contents differed not only in the emotional sequence but also in the storytelling and the framing. At the same time, this allowed us to investigate a situation very similar to what happens outside of laboratory contexts: consortiums for the protection of territorial products (or, in general, companies) rarely have to choose between creative proposals that differ in single separable variables; more often, they receive different proposals from several advertising agencies, and they need to choose those that have the highest probability of being remembered and generating functional emotions for the enhancement of their products. Although there are many practical implications of our approach, further basic research is needed for stronger support of our findings.

## 5. Conclusions

In this study, we compared two video commercials of traditional cheeses from Southern Italy using a neuromarketing approach in order to highlight the most effective one in terms of emotion and memorisation. Despite the fact that both the videos were composed of the same four narrative themes (i.e., territory, product, production techniques and natural landscapes), they differed in the emotional sequence: the first one was mainly characterised by a positive emotional tone, while the second one was characterised by an initial negative tone that turns to positive in the end. We found that the second video generates a better emotional reaction and memorisation than the first one. This is in line with the literature on charity advertising that showed how the negative–positive emotional sequence can boost the overall emotional perception and memorisation. Significant differences, however, emerged only when considering the videos as a whole and not when we compared individual themes, probably due to the difference in storytelling or in the personal characteristics of the sample. A future confirmatory study should verify these assumptions by fixing the storytelling while varying the emotional sequence, as well as taking into account gender and age as additional grouping factors. However, our results provide useful insights for those stakeholders who are engaged in the promotion of traditional agri-food products, especially small local realities, helping them to optimise investments and reduce the gap with the big food corporations. Effective communication should place emphasis on how the purchase of these products provides solutions to specific issues, rather than simply exalting the goodness of the products and the benefits associated with their consumption.

## Figures and Tables

**Table 1:** Descriptive statistics (M = mean, SD = standard deviation, n = number) of the MI, split for the video (M = The Myth, R = Rewind) and theme (Nt = Nature, Pr = Product, Prd = Production, Tr = Territoriality). All values are expressed as unitless z-scores.

Theme	Video	M	SD	n
Nt	M	0.001	0.251	18
	R	−0.015	0.687	17
Pr	M	−0.028	0.307	18
	R	−0.310	0.365	17
Prd	M	0.021	0.515	18
	R	−0.076	0.347	17
Tr	M	0.230	0.481	18
	R	−0.420	0.480	17

**Table 2:** Descriptive statistics (M = mean, SD = standard deviation, n = number) of the HR, split for the video (M = The Myth, R = Rewind) and theme (Nt = Nature, Pr = Product, Prd = Production, Tr = Territoriality). All values are expressed as unitless z-scores.

Theme	Video	M	SD	n
Nt	M	0.087	0.735	16
	R	−1.090	0.906	16
Pr	M	−0.310	0.538	16
	R	−0.970	0.866	16
Prd	M	0.316	0.513	16
	R	−1.081	0.803	16
Tr	M	0.232	1.241	16
	R	−0.324	1.514	16

**Table 3:** Descriptive statistics (M = mean, SD = standard deviation, n = number) of the EI, split for the video (M = The Myth, R = Rewind) and theme (Nt = Nature, Pr = Product, Prd = Production, Tr = Territoriality). All values are expressed as unitless z-scores.

Theme	Video	M	SD	n
Nt	M	0.005	0.178	16
	R	−0.255	0.345	17
Pr	M	−0.124	0.129	16
	R	−0.272	0.352	17
Prd	M	0.085	0.224	16
	R	−0.295	0.286	17
Tr	M	−0.084	0.273	16
	R	−0.280	0.581	17

**Table 4:** Summary of the six research hypotheses H1–H6 that compared AWI, MI, HR and EI metrics across both the Video (M = The Myth, R = Rewind) and the Video × Theme (Nt = Nature, Pr = Product, Prd = Production, Tr = Territoriality) dimensions. The direction of the expected differences is also provided, alongside the associated significant *p*-values (n.s. stands for not-significant). The fully or partially confirmed hypotheses are marked as, † and *, respectively.

Research Hypothesis	Metric	Direction	*p*-Value
H1 *	AWI	M > R	n.s.
	HR	M > R	0.045
	EI	M > R	0.009
H2	AWI	Nt${}_{M}$ > Nt${}_{R}$	n.s.
		Prd${}_{M}$ > Prd${}_{R}$	n.s.
		Tr${}_{M}$ > Tr${}_{R}$	n.s.
	HR	Nt${}_{M}$ > Nt${}_{R}$	n.s.
		Prd${}_{M}$ > Prd${}_{R}$	n.s.
		Tr${}_{M}$ > Tr${}_{R}$	n.s.
	EI	Nt${}_{M}$ > Nt${}_{R}$	n.s.
		Prd${}_{M}$ > Prd${}_{R}$	n.s.
		Tr${}_{M}$ > Tr${}_{R}$	n.s.
H3	AWI	Pr${}_{M}$ > Pr${}_{R}$	n.s.
	HR	Pr${}_{M}$ > Pr${}_{R}$	n.s.
	EI	Pr${}_{M}$ > Pr${}_{R}$	n.s.
H4 †	MI	M > R	0.025
H5 *	MI	Nt${}_{M}$ > Nt${}_{R}$	n.s.
		Prd${}_{M}$ > Prd${}_{R}$	n.s.
		Tr${}_{M}$ > Tr${}_{R}$	0.001
H6	MI	Pr${}_{M}$ > Pr${}_{R}$	n.s.

## Data Availability

All data are available upon request.

## Appendix A. Video Description

### Appendix A.1. Rewind

The video starts with a girl receiving a postal package that contains traditional cheeses from Southern Italy. The girl is shown so excited while unwrapping it that she decides to eat one of the products. At the moment of tasting, a scene change takes place, and the entire production process is shown in reverse (hence, the name Rewind), that is, from the end product to the raw materials. The main video sequences include the landscapes of Southern Italy, the animals grazing freely, the cow milking and the detailed cheese production phases—from the milk curdling to the cheese seasoning and marking.

### Appendix A.2. The Myth

The video starts with a voice-over narrating a legend (hence, the name The Myth). The Godhead gifted the human beings a harmonious and clean planet, the Hearth. Humans soon started harnessing nature, dealing with disastrous consequences. Several images of fires, natural landscapes polluted by garbage heaps and melting glaciers are shown. Displeased by their behaviour, the Godhead decides to punish them by erasing their memories. Five great sages decide to redeem humankind by starting the production of local cheeses using traditional techniques as a way to live in harmony with nature and preserve memories. Therefore, several images of natural landscapes, pictures of ancient villages in Southern Italy, animals grazing in pristine areas and cheeses produced with traditional techniques are shown.
